# Cooking or heating with solid fuels increased the all-cause mortality risk among mid-aged and elderly People in China

**DOI:** 10.1186/s12940-022-00903-6

**Published:** 2022-10-03

**Authors:** Yuxiang Yang, Yang Liu, Luolan Peng, Shuai Zhang, Changzheng Yuan, Wenyuan Li, Zuyun Liu, Yanan Ma

**Affiliations:** 1 NHC Key Laboratory of Trace Element Nutrition, National Institute for Nutrition and Health, Chinese Center for Disease Control and Prevention, 100050 Beijing, China; 2 Department of Biostatistics and Epidemiology, School of Public Health, China Medical University, No.77 Puhe Road, Shenyang North New Area, 110122 Shenyang, Liaoning China; 3 National Institute of Environmental Health, Chinese Center for Disease Control and Prevention, 100021 Beijing, China; 4 School of Public Health, Zhejiang University School of Medicine, 866 Yuhangtang Road, 310058 Hangzhou, Zhejiang China; 5 Department of Big Data in Health Science School of Public Health, Center for Clinical Big Data and Analytics Second Affiliated Hospital, Zhejiang University School of Medicine, 866 Yuhangtang Rd, 310058 Hangzhou, Zhejiang China

**Keywords:** Solid fuels, All-cause mortality, Mid-aged, Elderly

## Abstract

**Background:**

Our study aimed to explore the associations between solid fuels burning for either heating or cooking and all-cause mortality based on 2859 participants from the China Health and Retirement Longitudinal Study during 2011–2018.

**Methods:**

Logistic regression models were performed to estimate the risk for all-cause mortality between different types of fuels in the current longitudinal study. Furthermore, the combined impacts of applying solid fuels for both cooking and heating and the effect among those who switched types of fuels in cooking or heating during follow-up were also analyzed. Interaction and stratification analysis by covariables was applied further to explore the relationship between fuel burning and all-cause mortality.

**Results:**

After full-adjustment, usage of solid fuels was associated with higher all-cause mortality (for heating: OR = 1.93, 95% CI = 1.25, 3.00; for cooking: OR = 1.76, 95% CI = 1.10, 2.82). Using solid fuels for both cooking and heating (OR = 2.36; 95% CI, 1.38, 4.03) was associated with a higher risk of all-cause mortality, while using solid fuels with a single purpose was not (OR = 1.52; 95% CI, 0.90, 2.55). Protective tendencies were detected in switching solid to clean fuel for cooking (OR = 0.62; 95% CI, 0.32, 1.17) and heating (OR = 0.62; 95% CI, 0.35, 1.10).

**Conclusion:**

Either cooking or heating with solid fuels increases the risk of all-cause mortality among Chinese mid-aged and aging people in the urban area of China.

**Supplementary Information:**

The online version contains supplementary material available at 10.1186/s12940-022-00903-6.

## Introduction

Worldwide, around 40% of the population relies on burning solid fuel (e.g., biomass fuel, coal, and related fuel) for their household life, and the primary purpose is cooking and heating[1, 2]. Incomplete burning of these kinds of traditional fuels could release a great number of air pollutants, such as particulate matter of varying sizes, formaldehyde, carbon monoxide, nitrogen oxides, etc., which could lead to household air pollution (HAP)[3, 4]. In contrast, the combustion products from clean fuels (e.g., natural gas, electricity, and solar energy) are just water and carbon dioxide. It’s widely reported that HAP is currently one of the top ten risk factors for diseases including pneumonia, COPD, ischemic cardiovascular and cerebrovascular diseases, lung cancer, and cognitive decline, which may cause a heap of both social and health burdens all over the world[4–8].

Previous research was mainly conducted in low- and middle-income countries (LMICs)[9]. A global Burden of Disease (GBD) study reported that burning solid fuel for cooking had led to over 0.8 million Chinese premature death in 2010[10]. The Chinese Longitudinal Healthy Longevity Survey (CLHLS) showed that participants who used solid fuel for cooking had a 9% higher mortality risk than those who used clean fuels[4]. Meanwhile, researchers also reported that participants who switched fuels from solid to clean during follow-up didn’t show a significantly increased risk compared with those who stably used clean fuels[4]. Another study had estimated that solid fuel origin HAP had led to over 1 million Chinese premature mortalities in 2016[11]. Research from other regions like South Asia, Nigeria, and sub-Saharan Africa also showed that using solid cooking fuels could increase mortality among infants and children[12–14]. Thus, promoting the transition from solid fuel to clean fuel is not only a pathway toward improving global public health but also about human rights and environmental protection[15].

Only a few cohort studies have explored the associations of solid fuel-burning with all-cause mortality. A nationwide, large-scale cohort study found that using solid fuels for either heating or cooking would increase mortality risk; however, the participants were from only 5 rural areas in China[16, 17]. Although previous studies well-explored the association between solid fuel burning and health outcomes, there is still a lack of clear evidence to support the harmful effect caused by household fuel-burning based on the different HAP exposure patterns between cooking and heating within a nationwide sample[18], especially in the population of mid-aged or elderly who were more susceptible to chronic diseases.

Furthermore, little research focused on urban areas in this field. Although there is a huge gap between urban and rural under fuel modernization, many urban residents still use solid fuels in household life[11, 19]. Thus, we applied samples from China Health and Retirement Longitudinal Study (CHARLS) to explore the association between fuel for either cooking or heating and the risk of all-cause mortality among mid-aged or elderly participants in urban China. Meanwhile, we applied the potentially confounding effects of socioeconomic status and the house area (which might impact the ventilation function) and analyzed the interaction between fuels of different types. Moreover, the combined effect of applying solid fuels in both cooking and heating and the effect among those who switched fuels in cooking or heating during follow-up were also analyzed. Above all, we aim to give more authentic results about the relationship between burning solid fuels and all-cause mortality and may exert additional policies and regulations development in this field to achieve the public health goal.

## Methods

### Study design and participants

CHARLS is a national longitudinal cohort study covering 450 urban communities and rural villages across 28 provinces of China. Participants were all middle-aged and older adults. The research agenda of CHARLS has been described elsewhere in detail[20].

In a life history survey (CHARLS 2014), trained interviewers obtained the previous experience of each participant by a standard life history questionnaire. The survey offered the past information about the household fuels (coal, electricity, central heating, or gas) usage of 2011–2012 (Wave 1) nationally baseline participants.

**Fig. 1:**
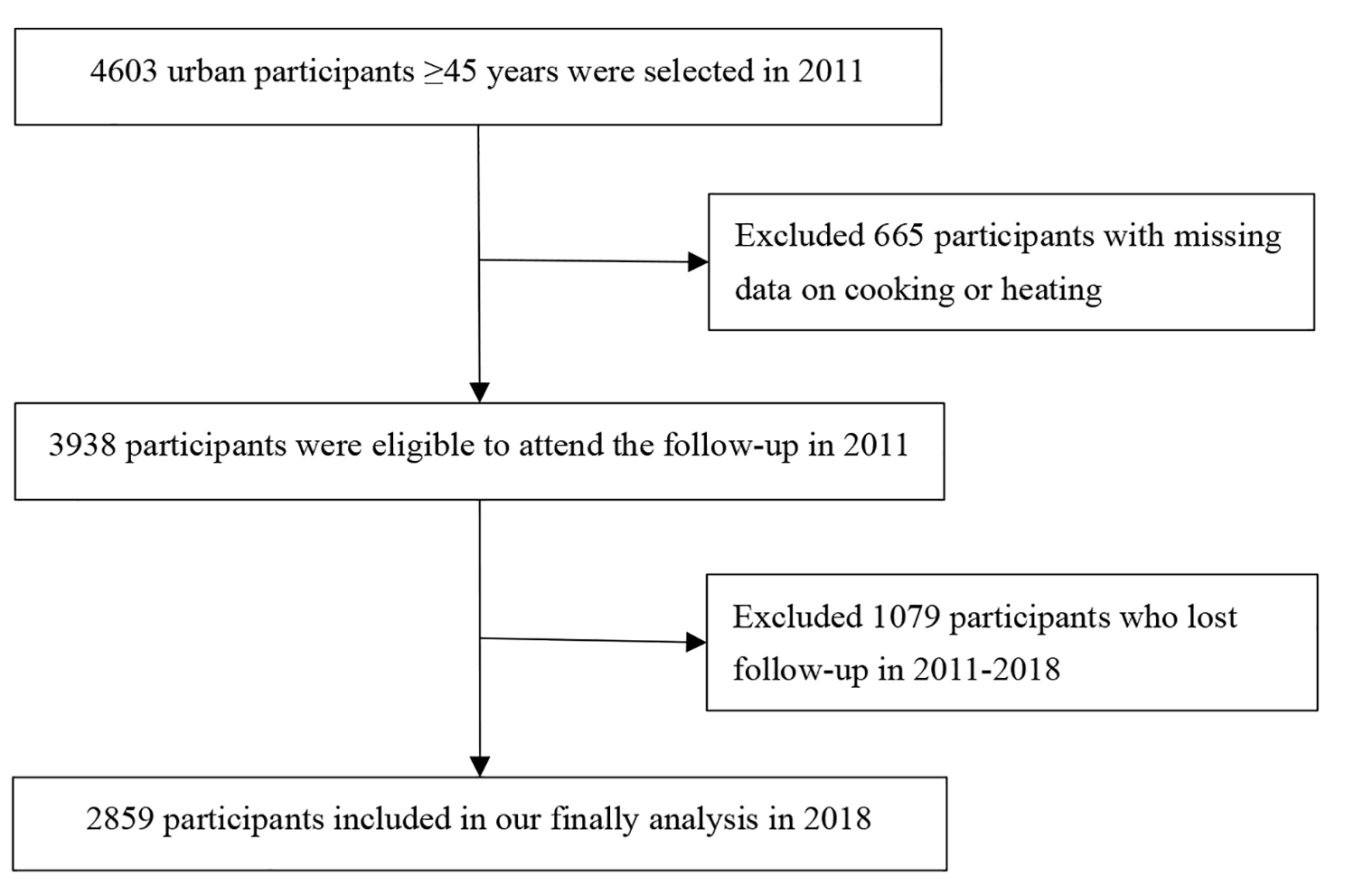
Flow diagram for participants enrolled in the study

Baseline survey was launched from 2011 to 2012 and included 4603 participants ≥ 45 years living in urban with follow-ups conducted every 2 or 3 years. As shown in Fig. 1, in this study, we excluded those participants who failed to report the type of household energy in 2011 (n = 665) and those who lost follow-up at the 7.5-year follow-up in 2018 (n = 1079). Eventually, a total of 2859 participants were included in the present study. The study protocol was approved by Peking University’s Ethical Review Committee (IRB 0000105211015). All participants wrote informed consent before participating in the study.

### Mortality status and follow-up

Data on mortality status were updated in 2011(Wave 1), 2013(Wave 2), 2014(Life history survey), 2015(Wave 3), 2018(Wave 4); the range of the follow-up period was about 8 years, which survival status via a field investigation. Trained interviewers investigated baseline survival status by a cover screen before being recruited in follow-up, and details of the field investigation are available online http://charls.pku.edu.cn/en. Database record all deaths occurring from April 2011 to March 2019, but the exact time of deaths were not recorded.

### Household energy source

The primary exposures were household energy sources, consisting of cooking and heating fuel types estimated by the following questions: ‘What is the main source of cooking fuel? (1) Coal, (2) Natural gas, (3) Marsh gas, (4) Liquefied petroleum gas, (5) electric, (6) Crop residue/Wood burning, (7) Other’, ‘Does your residence have heating? (1) Yes, (2) No’ and ‘What is the main heating energy source? (1) Solar, (2) Coal, (3) Natural gas, (4) Liquefied petroleum gas, (5) electric, (6) Crop residue/Wood burning, (7) Other’. Therefore, cooking fuels were divided into clean fuels (Natural gas, Marsh gas, Marsh gas, or electric) and solid fuels (coal, crop residue, wood burning). Likewise, heating fuels were categorized as clean fuels (uniform heating, solar, natural gas, liquefied petroleum gas, electric) and solid fuels (coal, crop residue, wood burning). We also assessed whether the fuel types were changed during the follow-up.

### Covariates

Covariates were selected in 2011 according to previous study [11]. Venous blood samples were collected and stored at -80 °C by medically trained staff from the Chinese Center for Disease Control and Prevention. Trained interviewers acquired data on age, sex, ethnicity, house area, marital status, annual household income, educational level, smoking status, drinking status, medical insurance status, and chronic diseases status via standard questionnaires. An electronic blood pressure monitor [20] was used by medical staff to measure participant blood pressure, with the mean of three measurements taken at 45 s intervals being recorded. Hypertension was defined as systolic blood pressure ≥ 140 mmHg and/or diastolic blood pressure ≥ 90 mmHg or usage of antihypertensive medicine[21]. Participants with self-reported diabetes, receiving diabetes treatment, meeting the American Diabetes Association (ADA) diabetes criteria (fasting plasma glucose ≥ 126 mg/dL or hemoglobin ≥ 6.5%) or participants with physician-diagnosed diabetes were defined as suffering from diabetes [22]. Body mass index (BMI) was calculated by dividing weight (kg) by height (m) squared. All but age and BMI were considered categorical variables in the current study. All the specific details about covariates above could be searched from the CHARLS website.

### Statistical analyses

Means ± standard deviations (SDs) and numbers (percentages) were used to describe continuous and categorical variables. Meanwhile, one-way ANOVA and Chi-square test were used to analyze differences between groups. Logistic regression models were applied to estimate the associations between household fuel types and all-cause mortality during the 7.5-years follow-up. Univariate and multivariate logistic regression analyses were utilized to investigate associations of factors (including types of household fuel, age, gender, ethnicity, BMI, house area, marital status, household annual income, education level, smoking status, drinking status, and medical insurance status) with all-cause mortality. Unadjusted and adjusted odds ratios (ORs) with 95% confidence intervals were calculated. Model I was adjusted for age and gender. Model II was adjusted for ethnicity, marital status, education level, annual household income, and medical insurance based on model 1. Model III also included drinking, smoking, BMI, house area, hypertension, and diabetes.

Furthermore, household fuel use was categorized into three levels (all clean fuels, mixed-use solid and clean fuels, and all solid fuels) based on the solid fuels that frequently occurred in cooking and heating. A full model was established to estimate the association. The self-reported switch from solid to clean fuels was also considered might twist the association between solid fuel use and all-cause mortality. Therefore, we further conducted a fully logistic regression model to check the association between fuel switch and all-cause mortality.

We then explored whether the associations differ by age, gender, ethnicity, BMI, house area, marital status, household annual income, education level, smoking status, drinking status, and medical insurance status by adding an interaction term. Age (≤ 60, > 60 years), gender (male, female), BMI (< 23, ≥ 23 kg/m^2^), house area (≤ 120, > 120 m^2^), marital status (live with spouse, live without spouse), household annual income (≤ 30,000, > 30,000 yuan), education level (< middle school, ≥middle school), smoking status (never smoker, ever smoker, current smoker), drinking status (never drinker, ever drinker, current drinker) and major chronic disease (no, yes) modified associations between household fuels and all-cause mortality. In addition, it was respectively used cooking fuels and heating fuels as effect modifiers with the full model to estimate the association between solid fuel use and deaths. Simultaneously, stratification analysis was also conducted to evaluate the effect of household fuels on outcomes occurring within 7.5 years follow-up further to confirm the association between household fuels and all-cause mortality. The robustness of results was confirmed after further excluding the participants who had cancer or CVD [23].

All statistical analyses were conducted using R (http://www.R-project.org; version 3.6.6) and EmpowerStats software (www.empowerstats.com, X&Y Solutions, Inc., Boston, MA, USA). A two-sided *P* value of < 0.05 was considered statistically significant.

## Results

### Basic characteristics of the study sample

The baseline characteristics of the participants recruited in the follow-up were shown according to the household fuel used in Tables 1 and 2, respectively. In sum, 2859 participants were enrolled in our final analysis. The distribution of age and gender of all 2859 participants in the follow-up analysis were 59.32 (10.22) years, and 1520 (53.18%) participants were female. Solid fuel was used for cooking and heating for around 587 (20.53%) and 998 (35.57%) participants, respectively. Further information is available in Table S1.

**Table 1:** Baseline characteristics of participants according to cooking fuels

Characteristic	Cooking fuels		
	**Clean fuels**	**Solid fuels**	***P*** **-value**
**N**	2272	587	
**Age(years)**	59.10 ± 10.11	60.16 ± 10.61	0.025
**BMI (kg/m** ^**2**^ **)**	24.77 ± 4.33	24.65 ± 3.86	0.592
**Gender, %**			0.828
Male	1066 (46.92%)	272 (46.42%)	
Female	1206 (53.08%)	314 (53.58%)	
**Ethnicity, %**			< 0.001
Other	130 (6.68%)	65 (12.65%)	
Han	1816 (93.32%)	449 (87.35%)	
**House area (m** ^**2**^ **)**			0.108
≤ 120	1832 (81.17%)	452 (78.20%)	
> 120	425 (18.83%)	126 (21.80%)	
**Marry status, %**			0.302
Live with spouse	1921 (84.70%)	487 (82.96%)	
Live without spouse	347 (15.30%)	100 (17.04%)	
**Household annual income (yuan)**			< 0.001
≤ 30,000	1189 (59.57%)	376 (75.65%)	
>30,000	807 (40.43%)	121 (24.35%)	
**Education level, %**			< 0.001
<Middle school	838 (37.03%)	363 (61.95%)	
≥Middle school	1425 (62.97%)	223 (38.05%)	
**Medical insurance status, %**			0.150
No	197 (8.71%)	62 (10.63%)	
Yes	2065 (91.29%)	521 (89.37%)	
**Smoking status, %**			0.020
Never smoker	1461 (66.35%)	351 (61.69%)	
Ever smoker	212 (9.63%)	49 (8.61%)	
Current smoker	529 (24.02%)	169 (29.70%)	
**Drinking status, %**			0.519
Never drinker	1360 (60.15%)	362 (61.88%)	
Ever drinker	167 (7.39%)	47 (8.03%)	
Current drinker	734 (32.46%)	176 (30.09%)	
**Hypertension, %**			0.916
No	1144 (50.35%)	297 (50.60%)	
Yes	1128 (49.65%)	290 (49.40%)	
**Diabetes, %**			0.971
No	1983 (87.28%)	512 (87.22%)	
Yes	289 (12.72%)	75 (12.78%)	

Values were means ± SD or n (percentages)

Values of polytomous variables may not sum to 100% due to rounding

**Table 2:** Baseline characteristics of participants according to heating fuels

Characteristic	Heating fuels		
	**Clean fuels**	**Solid fuels**	***P*** **-value**
**N**	1848	1011	
**Age(years)**	59.34 ± 10.27	59.29 ± 10.13	0.902
**BMI (kg/m** ^**2**^ **)**	24.85 ± 4.44	24.58 ± 3.89	0.147
**Gender, %**			0.928
Male	864 (46.75%)	474 (46.93%)	
Female	984 (53.25%)	536 (53.07%)	
**Ethnicity, %**			< 0.001
Other	88 (5.64%)	107 (11.88%)	
Han	1471 (94.36%)	794 (88.12%)	
**House area (m** ^**2**^ **)**			0.042
≤ 120	1502 (81.67%)	782 (78.51%)	
> 120	337 (18.33%)	214 (21.49%)	
**Marry status, %**			0.77
Live with spouse	1558 (84.49%)	850 (84.08%)	
Live without spouse	286 (15.51%)	161 (15.92%)	
**Household annual income (yuan)**			< 0.001
≤ 30,000	956 (58.12%)	609 (71.82%)	
>30,000	689 (41.88%)	239 (28.18%)	
**Education level, %**			< 0.001
<Middle school	641 (34.86%)	560 (55.45%)	
≥Middle school	1198 (65.14%)	450 (44.55%)	
**Medical insurance status, %**			< 0.001
No	134 (7.29%)	125 (12.41%)	
Yes	1704 (92.71%)	882 (87.59%)	
**Smoking status, %**			0.005
Never smoker	1200 (67.08%)	612 (62.32%)	
Ever smoker	174 (9.73%)	87 (8.86%)	
Current smoker	415 (23.20%)	283 (28.82%)	
**Drinking status, %**			0.119
Never drinker	1102 (59.99%)	620 (61.45%)	
Ever drinker	152 (8.27%)	62 (6.14%)	
Current drinker	583 (31.74%)	327 (32.41%)	
**Hypertension, %**			0.965
No	932 (50.43%)	509 (50.35%)	
Yes	916 (49.57%)	502 (49.65%)	
**Diabetes, %**			0.228
No	1623 (87.82%)	872 (86.25%)	
Yes	225 (12.18%)	139 (13.75%)	

Values were means ± SD or n (percentages)

Values of polytomous variables may not sum to 100% due to rounding

### Association between types of household fuel for cooking or heating and all-cause mortality

Table 3 showed the univariate associations between cooking fuels, heating fuels, age, gender, ethnicity, marital status, BMI, house area, household annual income, education level, smoking, drinking, or medical insurance status, and all-cause mortality. In univariate analysis, the association between cooking fuels or heating fuels and all-cause mortality was significant ([OR]: 1.71; 95% CI, 1.32, 2.22 and [OR]: 1.49; 95% CI, 1.17, 1.88, respectively).

**Table 3:** Univariate analysis between characteristics of participants with all-cause mortality

	Statistics	All-cause mortality
**Age(years)**	59.32 ± 10.22	1.12 (1.10, 1.13)
**BMI (kg/m** ^**2**^ **)**	24.74 ± 4.23	0.93 (0.90, 0.97)
**Cooking fuels, %**		
Clean fuels	2272 (79.47%)	1.0 (Reference)
Solid fuels	587 (20.53%)	1.71 (1.32, 2.22)
**Heating fuels, %**		
Clean fuels	1848 (64.64%)	1.0 (Reference)
Solid fuels	1011 (35.36%)	1.49 (1.17, 1.88)
**Gender, %**		
Male	1338 (46.82%)	1.0 (Reference)
Female	1520 (53.18%)	0.50 (0.40, 0.64)
**Ethnicity, %**		
Han	195 (7.93%)	1.0 (Reference)
Other	2265 (92.07%)	1.42 (0.78, 2.59)
**House area (m** ^**2**^ **)**		
≤ 120	2284 (80.56%)	1.0 (Reference)
> 120	551 (19.44%)	0.82 (0.60, 1.12)
**Marital status, %**		
Live with spouse	2408 (84.34%)	1.0 (Reference)
Live without spouse	447 (15.66%)	2.43 (1.86, 3.18)
**Household annual income (yuan)**		
≤ 30,000	1565 (62.78%)	1.0 (Reference)
>30,000	928 (37.22%)	0.82 (0.62, 1.07)
**Education level, %**		
<Middle school	1201 (42.16%)	1.0 (Reference)
≥Middle school	1648 (57.84%)	0.46 (0.37, 0.59)
**Medical insurance status, %**		
No	259 (9.10%)	1.0 (Reference)
Yes	2586 (90.90%)	0.81 (0.55, 1.18)
**Smoking status, %**		
Never smoker	1812 (65.39%)	1.0 (Reference)
Ever smoker	261 (9.42%)	2.70 (1.91, 3.81)
Current smoker	698 (25.19%)	1.61 (1.22, 2.12)
**Drinking status, %**		
Never drinker	1722 (60.51%)	1.0 (Reference)
Ever drinker	214 (7.52%)	2.19 (1.52, 3.17)
Current drinker	910 (31.97%)	0.98 (0.75, 1.28)
**Hypertension, %**		
No	1441 (50.40%)	1.0 (Reference)
Yes	1418 (49.60%)	2.52 (1.96, 3.23)
**Diabetes, %**		
No	2495 (87.27%)	1.0 (Reference)
Yes	364 (12.73%)	2.21 (1.65, 2.95)

Values were means ± SD or n (percentages)

Values of polytomous variables may not sum to 100% due to rounding

Table 4 shows the independent associations between solid fuel use for cooking and heating and all-cause mortality in the cohort. Solid (vs. clean) in cooking fuel users, the association remained robust after adjustment for age and gender in model I ( [OR]: 1.64; 95% CI, 1.23, 2.20), and further adjustment for ethnicity, marital status, education level, household annual income and medical insurance in model II ( [OR]: 1.62; 95% CI, 1.10, 2.40), as well as model III additionally adjustment for BMI, drinking status, smoking status, house area, hypertension, and diabetes [OR]: 1.76; 95% CI, 1.10, 2.82), while for heating, the ORs were [OR]: 1.64; 95% CI, 1.26, 2.13, [OR]: 1.52; 95% CI, 1.07, 2.17 and [OR]: 1.93; 95% CI, 1.25, 3.00 in Model I–III, respectively.

**Table 4:** Relationship between household fuels and risk of all-cause mortality in different models

Exposure		OR (95% CI)	
	Model I^a^	Model II^b^	Model III^c^
**Cooking fuels**			
Clean fuels	1.0 (Reference)	1.0 (Reference)	1.0 (Reference)
Solid fuels	1.64 (1.23, 2.20)	1.62 (1.10, 2.40)	1.76 (1.10, 2.82)
**Heating fuels**			
Clean fuels	1.0 (Reference)	1.0 (Reference)	1.0 (Reference)
Solid fuels	1.64 (1.26, 2.13)	1.52 (1.07, 2.17)	1.93 (1.25. 3.00)

Abbreviations: OR, odd ratio; CI, confidence interval

^a^ Adjust for Age (years) and Gender (Male, Female)

^b^ Adjust for Age (years), Gender (Male, Female), Ethnicity (Other, Han), Marital status (Live with spouse, Live without spouse), Education level (< Middle school, ≥Middle school), Household annual income (≤ 30,000, > 30,000) and Medical insurance (No, Yes)

^c^ Adjust for Age (years), BMI (kg/m^2^), Gender (Male, Female), Ethnicity (Other, Han), House area (≤ 120, > 120 m^2^), Marital status (Live with spouse, Live without spouse), Household annual income (≤ 30,000, > 30,000), Medical insurance (No, Yes), Education level (< Middle school, ≥Middle school), Smoking (Never smoker, Ever smoker, Current smoker), Drinking (Never drinker, Ever drinker, Current drinker), Hypertension (No, Yes), and Diabetes (No, Yes)

Table 5 found that double exposure to solid fuels for cooking and heating ([OR]: 2.36; 95% CI, 1.38, 4.03) was associated with a higher participants’ risk of all-cause mortality. Furthermore, using solid fuels for cooking or heating was not ([OR]: 1.52; 95% CI, 0.90, 2.55). Meanwhile, in Table 6 there was no significant difference in both switched solid fuel to clean fuel for cooking ([OR: 0.62; 95% CI, 0.32, 1.17) and heating ([OR]: 0.62; 95% CI,0.35, 1.10) but the same protective tendencies were detected in both.

**Table 5:** Adjusted Odd Ratio for all-cause mortality according to solid fuel use for cooking and heating

Exposure		OR (95% CI)	
	Model I^a^	Model II^b^	Model III^c^
All clean fuels	1.0 (Reference)	1.0 (Reference)	1.0 (Reference)
Mixed-use solid and clean fuels	1.31 (0.95, 1.80)	1.28 (0.84, 1.95)	1.52 (0.90, 2.55)
All solid fuels	1.96 (1.42, 2.71)	1.88 (1.21, 2.93)	2.36 (1.38, 4.03)

Abbreviations: OR, odd ratio; CI, confidence interval

^a^ Adjust for Age (years) and Gender (Male, Female)

^b^ Adjust for Age (years), Gender (Male, Female), Ethnicity (Other, Han), Marital status (Live with spouse, Live without spouse), Education level (< Middle school, ≥Middle school), Household annual income (≤ 30,000, > 30,000) and Medical insurance (No, Yes)

^c^ Adjust for Age (years), BMI (kg/m^2^), Gender (Male, Female), Ethnicity (Other, Han), House area (≤ 120, > 120 m^2^), Marital status (Live with spouse, Live without spouse), Household annual income (≤ 30,000, > 30,000), Medical insurance (No, Yes), Education level (< Middle school, ≥Middle school), Smoking (Never smoker, Ever smoker, Current smoker), Drinking (Never drinker, Ever drinker, Current drinker), Hypertension (No, Yes), and Diabetes (No, Yes)

**Table 6:** Adjusted Odd Ratio for all-cause mortality in association with previous switch from solid to clean fuels

Exposure		OR (95% CI)	
	Model I^a^	Model II^b^	Model III^c^
**Previous switch from solid to clean fuels**			
Cooking			
Solid fuel use	1.0 (Reference)	1.0 (Reference)	1.0 (Reference)
Solid to clean fuel use	0.53 (0.33, 0.84)	0.59 (0.35, 1.01)	0.62 (0.32, 1.17)
Heating			
Solid fuel use	1.0 (Reference)	1.0 (Reference)	1.0 (Reference)
Solid to clean fuel use	0.53 (0.36, 0.79)	0.59 (0.37, 0.94)	0.62 (0.35, 1.10)

Abbreviations: OR, odd ratio; CI, confidence interval

^a^ Adjust for Age (years) and Gender (Male, Female)

^b^ Adjust for Age (years), Gender (Male, Female), Ethnicity (Other, Han), Marital status (Live with spouse, Live without spouse), Education level (< Middle school, ≥Middle school), Household annual income (≤ 30,000, > 30,000) and Medical insurance (No, Yes)

^c^ Adjust for Age (years), BMI (kg/m^2^), Gender (Male, Female), Ethnicity (Other, Han), House area (≤ 120, > 120 m^2^), Marital status (Live with spouse, Live without spouse), Household annual income (≤ 30,000, > 30,000), Medical insurance (No, Yes), Education level (< Middle school, ≥Middle school), Smoking (Never smoker, Ever smoker, Current smoker), Drinking (Never drinker, Ever drinker, Current drinker), Hypertension (No, Yes), and Diabetes (No, Yes)

In Table S2 and Table S3, there were no interactions between cooking fuels and heating fuels (*P* for interaction > 0.05). The results revealed that although the interaction of solid fuels from cooking and heating was not modified the association between solid fuels and all-cause mortality, the strengthen hazard effect was detected. When we further excluded the participants who had malignant cancer or CVD, the association between solid fuels and death remained in Table S4. In addition, interaction tests and stratified results for characteristics were shown in Table S5 and Table S6.

## Discussion

The current longitudinal study indicated that using solid fuels for heating or cooking was positively associated with all-cause mortality after multiple adjustments. Afterwards, the association between burning solid fuels and the risk of all-cause mortality by different usage categories (all clean fuels, mixed-use solid and clean fuels, and all solid fuels) was also evaluated. It showed that compared with clean fuels’ users, using solid fuels for both heating and cooking had a higher risk of all-cause mortality. Furthermore, regarding the condition of changing fuel’s type, protective tendencies of all-cause mortality were observed among those who switched the fuels from solid to clean compared with those stable solid fuels’ users.

They were accompanied by the rapid development of the national economy and power infrastructure, more and more people selected clean fuels as an alternative energy source[19, 24]. However, as the biggest developing country globally and one of the LMICs, China remains heavily dependent on solid fuels [11, 19]. Besides, it’s known that people are more vulnerable to air pollutants with age increasing[25]. Moreover, there is a tendency that nowadays, more and more Chinese people are willing to move to the countryside after retirement or under poor health conditions, which indicates a significant intervention point to raise the awareness among this group of people to utilize clean instead of solid fuels[4, 26]. Our findings may provide a beneficial pathway for improving health conditions and prolonging life.

This study found that burning solid fuel was positively associated with the risk of all-cause mortality. The results also showed that those who switched from solid fuels to cleaning might not have a higher risk than those who stably used clean fuels. Simultaneously using solid fuels, cooking, and heating would further increase the risk of all-cause mortality compared to those who use clean fuels.

The same results were obtained in previous studies. A prospective study conducted in China among 13,528 non-smokers to explore the relationship between heating fuel types and all-cause mortality showed that solid fuel heating could indicate a 55% higher risk of all-cause mortality compared with participants who used clean fuels. It also increased the risk of stroke among non-smokers[27]. Another research by Liu et al. showed that indoor solid fuel combustion for heating might increase the risk of cervical cancer death after full adjustment[28]. There was another report that, unlike those using clean fuels for heating, solid fuels were positively associated with cardiovascular mortality and all-cause mortality. Meanwhile, participants who reported having previously switched to clean fuels for cooking had a lower cardiovascular and all-cause mortality[17].

As for studies focused on the relationship between cooking fuels and mortality, our study results were consistent with previous research. For instance, a national retrospective longitudinal survey among Chinese showed that when analyzing the relationship between cooking fuels and the risk for all-cause mortality, those using solid fuels had 1.09 times higher risk of all-cause mortality than those using clean fuels. Also, significantly increased risk was not observed in those who switched from solid fuels to clean ones during the follow-up period, which indicated the need to promote fuel reform[4]. Findings from urban participants enrolled in China Kadoorie Biobank (CKB) showed that persistent solid fuels’ users had 19% higher risks of all-cause mortality. Besides, compared with the participants who used clean cooking fuels, solid ones also had a higher risk of 24% and 43% mortality caused by cardiovascular diseases and respiratory diseases, respectively[16]. In another study, using solid fuels for cooking was associated with a greater risk (over 10% for both). Participants who transferred to clean fuels had a lower risk for both mortalities than persistent solid fuel users[17].

This study also examined the joint effects of using solid fuels for cooking and heating. To our best knowledge, no previous research had focused on this part. Results indicated that using solid fuels for both purposes would over double the risk of all-cause mortality compared to using clean fuels for cooking and heating. It may be explained that the former would increase the exposing time of HAP and was always accompanied by poor ventilation in those who use solid fuels in the household[6]. Moreover, we discussed the effect among those who switched the household burning fuels from solid to clean one, and it showed that there was no significant difference in both switched solid fuels to clean fuels for either cooking or heating, but the same protective tendencies were detected in both.

Several previous studies explained the adverse effects caused by burning solid fuels. Air pollutants, such as carbon monoxide, sulfur dioxide, nitrogen oxide, polycyclic aromatic hydrocarbons, particulate matter, and heavy metals, are always released by an uncompleted combustion process[29, 30]. These pollutants link to activate various physiological processes, i.e., the production of reactive oxygen species (ROS), oxidative stress, and systemic inflammation[31], as well as causing further damage by disrupting human proteins, esters, and DNA and causing oxidative damage[32]. Afterward, the above process with long-term exposure might initiate several health impairments and related diseases on cardiovascular, cognitive, respiratory, and other human structures and functions[5, 33–35].

Based on the current results, although the government has made great efforts to promote fuel revolution in the past decades[36], there is still a neglectable proportion of urban residents who were still using solid fuels in their household life, which did lie a huge burden on health and well-being. It could partly be explained that there are a lot of “urban villages” currently in China, people who live here tend to have a poor environment and public health safety[37]. Thus, future fuel improvement policies should still pay more attention to people who live in urban areas, especially in urban villages, not only in rural areas. Moreover, except changing the types of fuels, techniques for making advanced solid fuels and indoor-ventilate could also be considered to address and assist in solving the problem of HAP. An interesting report showed that, in high-income countries like countries in Europe and North America, although more and more people chose solid fuels for heating, they experienced less HAP due to sophisticated fuel tech and pollutant discharge[38]. In summary, under the remarkable aging process in China, there is an urgent need to take various measures including those mentioned above to release the burden on the health system. Decreasing the exposure to HAP by burning solid fuels could be applied as one of the crucial parts and could be validated by longitudinal or interventional studies in the future research if it’s possible.

Apart from the study design, our study also has several other strengths. Except for long-term follow-up, our study explored the adverse effects caused by either cooking fuels or heating fuels, fitting multiple covariates (i.e., ethnic, house area) that hadn’t been broadly considered in the previous studies into multiple adjustments. The results might provide more solid evidence of the relationship between burning fuels and all-cause mortality. However, our study did have some limitations. Firstly, due to the inherent limitations of the prospective study, the loss of follow-up was inevitable, and some changes like the shifting type of fuels in household life may happen during the long-term follow-up intervals. However, this study had a relatively intensive frequency of follow-up. Secondly, the condition of household ventilation and stoves that may impact our outcomes was unavailable due to the characteristics of the database[17]. Thirdly, our study didn’t adjust the effect of outdoor pollutants, physical activity, and confounding covariates, so the possibility of residual confounding, unmeasured confounding, and measurement error persists. These limitations should be mended in further research if it’s available.

## Conclusion

Our current study found that burning solid fuel was positively associated with the risk of all-cause mortality regardless of the purpose for either cooking or heating. Plus, using solid fuels for cooking and heating would double the risk of all-cause mortality compared to those who singly use clean fuels. The above results may also serve as a direction for detecting specific intervention groups contributing to more accurate prevention services. Further research may target to potential effects of using different household fuels.

## Electronic supplementary material

Below is the link to the electronic supplementary material.

Supplementary Material 1

## Data Availability

All the data and materials were based on China Health and Retirement Longitudinal Study conducted from 2011 to 2018. Available: http://charls.pku.edu.cn/.

## Abbreviations

BMI: Body mass index
CHARLS: China health and retirement longitudinal study
CI: Confidence interval
CLHLS: Chinese longitudinal healthy longevity Survey
COPD: Chronic obstructive pulmonary diseases
GBD: Global burden of disease
HAP: Household air pollution
LMICs: Low- and middle-income countries
OR: Odds ratio
ROS: Reactive oxygen species
SD: Standard deviation
