# Supplementary material for: Cooking or heating with solid fuels increased the all-cause mortality risk among mid-aged and elderly People in China
Source: Environ Health. 2022 Oct 3;21:91. doi: 10.1186/s12940-022-00903-6 (PMC9528092; doi:10.1186/s12940-022-00903-6)
Supplement: Supplementary file 1 — Supplementary Material 1 [file 12940_2022_903_MOESM1_ESM.docx]

Table S1. Baseline characteristics of participants according to survival status

| **Characteristic** | **Survival status** | |  |
| --- | --- | --- | --- |
|  | **Survival** | **Death** | ***P*-value** |
| N | 2541 | 318 |  |
| Age(years) | 58.02 ± 9.38 | 69.70 ± 10.73 | <0.001 |
| BMI (kg/m^2^) | 24.85 ± 4.22 | 23.79 ± 4.22 | <0.001 |
| Cooking fuels, % |  |  | <0.001 |
| Clean fuel | 2047 (80.56%) | 225 (70.75%) |  |
| Solid fuel | 494 (19.44%) | 93 (29.25%) |  |
| Heating fuels, % |  |  | <0.001 |
| Clean fuel | 1669 (65.68%) | 179 (56.29%) |  |
| Solid fuel | 872 (34.32%) | 139 (43.71%) |  |
| Gender, % |  |  | <0.001 |
| Male | 1142 (44.94%) | 196 (61.83%) |  |
| Female | 1399 (55.06%) | 121 (38.17%) |  |
| Ethnicity, % |  |  | 0.251 |
| Other | 183 (8.12%) | 12 (5.85%) |  |
| Han | 2072 (91.88%) | 193 (94.15%) |  |
| House area (m^2^) |  |  | 0.204 |
| ≤120 | 2021 (80.23%) | 263 (83.23%) |  |
| >120 | 498 (19.77%) | 53 (16.77%) |  |
| Marry status, % |  |  | <0.001 |
| Live with spouse | 2182 (85.94%) | 226 (71.52%) |  |
| Live without spouse | 357 (14.06%) | 90 (28.48%) |  |
| Household annual income (yuan) |  |  | 0.137 |
| ≤30000 | 1385 (62.28%) | 180 (66.91%) |  |
| ＞30000 | 839 (37.72%) | 89 (33.09%) |  |
| Education level, % |  |  | <0.001 |
| <Middle school | 1015 (40.06%) | 186 (59.05%) |  |
| ≥Middle school | 1519 (59.94%) | 129 (40.95%) |  |
| Medical insurance status, % |  |  | 0.269 |
| No | 225 (8.89%) | 34 (10.79%) |  |
| Yes | 2305 (91.11%) | 281 (89.21%) |  |
| Smoking status, % |  |  | <0.001 |
| Never smoker | 1659 (67.00%) | 153 (51.86%) |  |
| Ever smoker | 209 (8.44%) | 52 (17.63%) |  |
| Current smoker | 608 (24.56%) | 90 (30.51%) |  |
| Drinking status, % |  |  | <0.001 |
| Never drinker | 1545 (60.97%) | 177 (56.73%) |  |
| Ever drinker | 171 (6.75%) | 43 (13.78%) |  |
| Current drinker | 818 (32.28%) | 92 (29.49%) |  |
| Hypertension, % |  |  | <0.001 |
| No | 1343 (52.85%) | 98 (30.82%) |  |
| Yes | 1198 (47.15%) | 220 (69.18%) |  |
| Diabetes, % |  |  | <0.001 |
| No | 2248 (88.47%) | 247 (77.67%) |  |
| Yes | 293 (11.53%) | 71 (22.33%) |  |

Values were means ± SD or n (percentages).

Values of polytomous variables may not sum to 100% due to rounding.

Table S2. Stratified analyses of between solid fuel use for cooking and all-cause mortality^a^

| **Exposure** | **Cooking fuels, OR (95% CI)** | |  |
| --- | --- | --- | --- |
|  | **Clean fuels** | **Solid fuels** | ***P* for interaction** |
| **Heating fuels** |  |  | 0.616 |
| Clean fuel (N=1848) | 1.0 (Reference) | 0.99 (0.30, 3.29) |  |
| Solid fuel (N=1011) | 1.0 (Reference) | 1.53 (0.83, 2.81) |  |

Abbreviations: OR, odd ratio; CI, confidence interval.

**^a^** Adjust for Age (years), BMI (kg/m^2^), Gender (Male, Female), Ethnicity (Other, Han), House area (≤120, >120 m^2^), Marital status (Live with spouse, Live without spouse), Household annual income (≤30000, >30000), Medical insurance (No, Yes), Education level (<Middle school, ≥Middle school), Smoking (Never smoker, Ever smoker, Current smoker), Drinking (Never drinker, Ever drinker, Current drinker), Hypertension (No, Yes), and Diabetes (No, Yes).

Table S3. Stratified analyses of between solid fuel use for heating and all-cause mortality^a^

| **Exposure** | **Heating fuels, OR (95% CI)** | |  |
| --- | --- | --- | --- |
|  | **Clean fuels** | **Solid fuels** | ***P* for interaction** |
| **Cooking fuels** |  |  | 0.616 |
| Clean fuel (N=2272) | 1.0 (Reference) | **1.97 (1.11, 3.49)** |  |
| Solid fuel (N=587) | 1.0 (Reference) | 2.05 (0.63, 6.68) |  |

Abbreviations: OR, odd ratio; CI, confidence interval.

**^a^** Adjust for Age (years), BMI (kg/m^2^), Gender (Male, Female), Ethnicity (Other, Han), House area (≤120, >120 m^2^), Marital status (Live with spouse, Live without spouse), Household annual income (≤30000, >30000), Medical insurance (No, Yes), Education level (<Middle school, ≥Middle school), Smoking (Never smoker, Ever smoker, Current smoker), Drinking (Never drinker, Ever drinker, Current drinker), Hypertension (No, Yes), and Diabetes (No, Yes).

Table S4. Relationship between household fuels and risk of all-cause mortality in different models after further excluding the participants with CVD and malignant cancer at baseline

| **Exposure** |  | **OR (95% CI)** |  |
| --- | --- | --- | --- |
|  | **Model I^a^** | **Model II^b^** | **Model III^c^** |
| **Cooking fuels** |  |  |  |
| Clean fuels (N=1785) | 1.0 (Reference) | 1.0 (Reference) | 1.0 (Reference) |
| Solid fuels (N=438) | 1.36 (0.94, 1.97) | 1.34 (0.80, 2.25) | 1.37 (0.73, 2.57) |
| **Heating fuels** |  |  |  |
| Clean fuels (N=1472) | 1.0 (Reference) | 1.0 (Reference) | 1.0 (Reference) |
| Solid fuels (N=751) | 1.68 (1.22, 2.33) | 1.59 (1.01, 2.50) | 1.94 (1.10, 3.43) |

Abbreviations: OR, odd ratio; CI, confidence interval.
^a^ Adjust for Age (years) and Gender (Male, Female).
^b^ Adjust for Age (years), Gender (Male, Female), Ethnicity (Other, Han), Marital status (Live with spouse, Live without spouse), Education level (<Middle school, ≥Middle school), Household annual income (≤30000, >30000) and Medical insurance (No, Yes).
^c^ Adjust for Age (years), BMI (kg/m^2^), Gender (Male, Female), Ethnicity (Other, Han), House area (≤120, >120 m^2^), Marital status (Live with spouse, Live without spouse), Household annual income (≤30000, >30000), Medical insurance (No, Yes), Education level (<Middle school, ≥Middle school), Smoking (Never smoker, Ever smoker, Current smoker), Drinking (Never drinker, Ever drinker, Current drinker), Hypertension (No, Yes), and Diabetes (No, Yes).

Table S5. Stratified analyses of between cooking fuels and risk of all-cause mortality^a^

| **Exposure** | **Cooking fuels, OR (95% CI)** | |  |
| --- | --- | --- | --- |
|  | **Clean fuels** | **Solid fuels** | ***P* for interaction** |
| Age |  |  | 0.188 |
| ≤60 (N=1690) | 1.0 (Reference) | 3.39 (1.41, 8.13) |  |
| >60 (N=1168) | 1.0 (Reference) | 1.42 (0.82, 2.46) |  |
| Gender |  |  | 0.043 |
| Male (N=1338) | 1.0 (Reference) | 2.71 (1.47, 5.00) |  |
| Female (N=1520) | 1.0 (Reference) | 0.99 (0.45, 2.18) |  |
| BMI (kg/m^2^) |  |  | 0.319 |
| <23 (N=684) | 1.0 (Reference) | 1.23 (0.58, 2.60) |  |
| ≥23 (N=1333) | 1.0 (Reference) | 2.04 (1.10, 3.79) |  |
| House area (m^2^) |  |  | 0.848 |
| ≤120 (N=2284) | 1.0 (Reference) | 1.76 (1.03, 3.01) |  |
| >120 (N=551) | 1.0 (Reference) | 1.74 (0.56, 5.41) |  |
| Marital status |  |  | 0.013 |
| Live with spouse (N=2408) | 1.0 (Reference) | 2.35 (1.37, 4.01) |  |
| Live without spouse (N=447) | 1.0 (Reference) | 0.67 (0.22, 2.03) |  |
| Household annual income (yuan) |  |  | 0.136 |
| ≤30000 (N=1565) | 1.0 (Reference) | 2.09 (1.23, 3.54) |  |
| ＞30000 (N=928) | 1.0 (Reference) | 0.73 (0.22, 2.48) |  |
| Education level |  |  | 0.315 |
| <Middle school (N=1201) | 1.0 (Reference) | 1.56 (0.84, 2.89) |  |
| ≥Middle school (N=1648) | 1.0 (Reference) | 2.45 (1.15, 5.23) |  |
| Smoking status |  |  | 0.043 |
| Never smoker (N=1812) | 1.0 (Reference) | 0.91 (0.43, 1.94) |  |
| Ever smoker (N=261) | 1.0 (Reference) | 1.59 (0.37, 6.90) |  |
| Current smoker (N=698) | 1.0 (Reference) | 3.86 (1.72, 8.63) |  |
| Drinking status |  |  | 0.312 |
| Never drinker (N=1722) | 1.0 (Reference) | 1.21 (0.61, 2.38) |  |
| Ever drinker (N=214) | 1.0 (Reference) | 5.01 (0.59, 42.62) |  |
| Current drinker (N=910) | 1.0 (Reference) | 2.52 (1.15, 5.52) |  |
| Major chronic disease |  |  | 0.611 |
| No (N=1318) | 1.0 (Reference) | 1.49 (0.64, 3.45) |  |
| Yes (N=1541) | 1.0 (Reference) | 1.83 (1.03, 3.25) |  |

Abbreviations: OR, risk ratio; CI, confidence interval.

**^a^** Adjust for Age (years), BMI (kg/m^2^), Gender (Male, Female), Ethnicity (Other, Han), House area (≤120, >120 m^2^), Marital status (Live with spouse, Live without spouse), Household annual income (≤30000, >30000), Medical insurance (No, Yes), Education level (<Middle school, ≥Middle school), Smoking (Never smoker, Ever smoker, Current smoker), Drinking (Never drinker, Ever drinker, Current drinker), Hypertension (No, Yes), and Diabetes (No, Yes). Of note, variables examined in this table were not adjusted.

Table S6. Stratified analyses of between heating fuels and risk of all-cause mortality^a^

| **Exposure** | **Heating fuels, OR (95% CI)** | |  |
| --- | --- | --- | --- |
|  | **Clean fuels** | **Solid fuels** | ***P* for interaction** |
| Age |  |  | 0.828 |
| ≤60 (N=1690) | 1.0 (Reference) | 2.28 (0.93, 5.55) |  |
| >60 (N=1168) | 1.0 (Reference) | 1.72 (1.05, 2.83) |  |
| Gender |  |  | 0.006 |
| Male (N=1338) | 1.0 (Reference) | 3.49 (1.90, 6.38) |  |
| Female (N=1520) | 1.0 (Reference) | 0.87 (0.43, 1.80) |  |
| BMI (kg/m^2^) |  |  | 0.600 |
| <23 (N=684) | 1.0 (Reference) | 2.61 (1.26, 5.40) |  |
| ≥23 (N=1333) | 1.0 (Reference) | 1.70 (0.97, 3.01) |  |
| House area (m^2^) |  |  | 0.507 |
| ≤120 (N=2284) | 1.0 (Reference) | 2.12 (1.29, 3.48) |  |
| >120 (N=551) | 1.0 (Reference) | 1.05 (0.35, 3.14) |  |
| Marital status |  |  | 0.001 |
| Live with spouse (N=2408) | 1.0 (Reference) | 2.81 (1.67, 4.73) |  |
| Live without spouse (N=447) | 1.0 (Reference) | 0.68 (0.25, 1.86) |  |
| Household annual income (yuan) |  |  | 0.345 |
| ≤30000 (N=1565) | 1.0 (Reference) | 2.08 (1.23, 3.53) |  |
| ＞30000 (N=928) | 1.0 (Reference) | 1.57 (0.65, 3.78) |  |
| Education level |  |  | 0.065 |
| <Middle school (N=1201) | 1.0 (Reference) | 1.41 (0.78, 2.54) |  |
| ≥Middle school (N=1648) | 1.0 (Reference) | 3.09 (1.58, 6.04) |  |
| Smoking status |  |  | 0.059 |
| Never smoker (N=1812) | 1.0 (Reference) | 1.13 (0.59, 2.16) |  |
| Ever smoker (N=261) | 1.0 (Reference) | 3.35 (0.96, 11.73) |  |
| Current smoker (N=698) | 1.0 (Reference) | 3.94 (1.65, 9.39) |  |
| Drinking status |  |  | 0.074 |
| Never drinker (N=1722) | 1.0 (Reference) | 1.17 (0.64, 2.16) |  |
| Ever drinker (N=214) | 1.0 (Reference) | 23.49 (1.70, 324.02) |  |
| Current drinker (N=910) | 1.0 (Reference) | 2.71 (1.25, 5.91) |  |
| Major chronic disease |  |  | 0.799 |
| No (N=1318) | 1.0 (Reference) | 1.91 (0.87, 4.20) |  |
| Yes (N=1541) | 1.0 (Reference) | 1.93 (1.14, 3.27) |  |

Abbreviations: OR, risk ratio; CI, confidence interval.

**^a^** Adjust for Age (years), BMI (kg/m^2^), Gender (Male, Female), Ethnicity (Other, Han), House area (≤120, >120 m^2^), Marital status (Live with spouse, Live without spouse), Household annual income (≤30000, >30000), Medical insurance (No, Yes), Education level (<Middle school, ≥Middle school), Smoking (Never smoker, Ever smoker, Current smoker), Drinking (Never drinker, Ever drinker, Current drinker), Hypertension (No, Yes), and Diabetes (No, Yes). Of note, variables examined in this table were not adjusted.
